# Morin Hydrate Encapsulation and Release from Mesoporous Silica Nanoparticles for Melanoma Therapy

**DOI:** 10.3390/molecules28124776

**Published:** 2023-06-15

**Authors:** Catarina Cunha, Diogo Marinheiro, Bárbara J. M. L. Ferreira, Helena Oliveira, Ana L. Daniel-da-Silva

**Affiliations:** 1 Department of Biology, CESAM-Centre for Environmental and Marine Studies, University of Aveiro, 3810-193 Aveiro, Portugal; catarinafilipacunha@ua.pt; 2 Department of Chemistry, CICECO-Aveiro Institute of Materials, University of Aveiro, 3810-193 Aveiro, Portugal; diogomarinheiro@ua.pt (D.M.); barbaraferreira@ua.pt (B.J.M.L.F.)

**Keywords:** melanoma, morin, mesoporous silica nanoparticles, nanotechnology, nano delivery system

## Abstract

Melanoma incidence, a type of skin cancer, has been increasing worldwide. There is a strong need to develop new therapeutic strategies to improve melanoma treatment. Morin is a bioflavonoid with the potential for use in the treatment of cancer, including melanoma. However, therapeutic applications of morin are restrained owing to its low aqueous solubility and limited bioavailability. This work investigates morin hydrate (MH) encapsulation in mesoporous silica nanoparticles (MSNs) to enhance morin bioavailability and consequently increase the antitumor effects in melanoma cells. Spheroidal MSNs with a mean size of 56.3 ± 6.5 nm and a specific surface area of 816 m^2^/g were synthesized. MH was successfully loaded (MH-MSN) using the evaporation method, with a loading capacity of 28.3% and loading efficiency of 99.1%. In vitro release studies showed that morin release from MH-MSNs was enhanced at pH 5.2, indicating increased flavonoid solubility. The in vitro cytotoxicity of MH and MH-MSNs on human A375, MNT-1 and SK-MEL-28 melanoma cell lines was investigated. Exposure to MSNs did not affect the cell viability of any of the cell lines tested, suggesting that the nanoparticles are biocompatible. The effect of MH and MH-MSNs on reducing cell viability was time- and concentration-dependent in all melanoma cell lines. The A375 and SK-MEL-28 cell lines were slightly more sensitive than MNT-1 cells in both the MH and MH-MSN treatments. Our findings suggest that MH-MSNs are a promising delivery system for the treatment of melanoma.

## 1. Introduction

The incidence of cancer is rising worldwide and is one of the most common causes of mortality, being a major global public health concern [1,2,3]. Melanoma skin cancer is very common in Caucasians and arises due to genetically altered melanocytes, cells responsible for melanin production [4,5,6,7]. Genetic propensity is one of the causes of melanoma, in addition to sun exposure [3,8]. UVB radiation can cause direct DNA damage and alter the DNA methylation profile of skin cells, which may contribute to genomic instability, usually associated with repetitive sequence dysregulation, and consequently leads to the development of skin cancer in humans [8,9,10,11,12,13].

Traditional melanoma treatments include surgery, mostly used in the early stages, and in metastatic stages, chemotherapy, radiotherapy, targeted therapy and immunotherapy are the chosen therapeutic strategies [14,15]. This type of skin cancer is very aggressive since cancer cells present uncontrolled and abnormal proliferation, triggering metastasis, which makes traditional treatments poorly effective [16,17]. Additionally, the tumor heterogenicity and drug resistance shown by tumor cells, which are mutually related, are another obstacle to treatment success [18,19].

A decline in death rates for melanoma was reported in the United States between 2013 and 2017 after the introduction of new therapies for metastatic melanoma [1], showing that the development of new therapeutic strategies is essential to treat melanoma.

**Figure 1:**
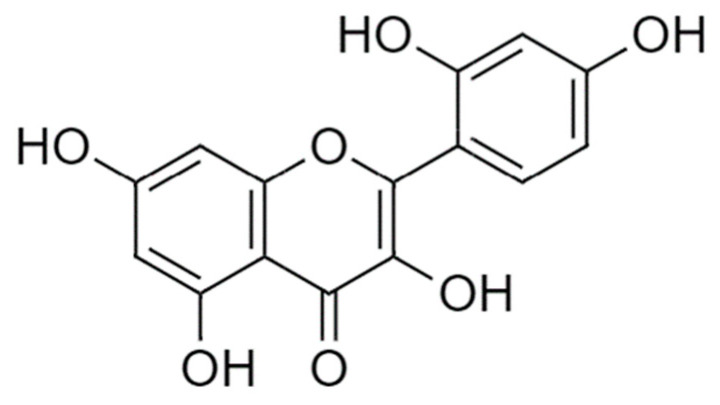
Morin chemical structure.

The use of novel compounds in melanoma treatment using a nanoscale approach represents a promising strategy to overcome the current limitations of conventional therapies. Flavonoids, a group of polyphenolic compounds mainly found in plants, fruits and vegetables, have demonstrated potential anticancer capacity [20,21,22]. Several studies have shown that flavonoids inhibited cell proliferation and invasion and induced apoptosis [23,24,25,26,27] through different mechanisms, such as the regulation of the cell cycle and cellular metabolism, and epigenetic modification, such as DNA methylation [4,28,29]. One such flavonoid is morin (3,5,7,2′,4′-pentahydroxyflavone) (Figure 1), which is commonly found in a variety of fruits and vegetables, including figs, mulberries, apples and onions [30,31]. Morin has demonstrated anticancer properties against different types of cancer including melanoma [30,32,33]. Morin possesses strong antioxidant properties due to its capacity to scavenge free radicals [34]. It promotes cell cycle arrest and inhibits cancer cell proliferation and invasion by suppressing proteins involved in cell cycle regulation and by blocking the synthesis of DNA [35,36,37,38]. Moreover, morin induces apoptosis in cancer cells via several mechanisms, such as activating caspase -8, -9 and -3 or by suppressing the nuclear factor-ĸB [35,36,39,40,41,42]. Additionally, morin could be used as an adjuvant for conventional chemotherapeutic drugs. For instance, Chung et al. showed that the combination of morin with telomerase inhibitor MST-312 sensitized resistant tumor cells and consequently increased the efficacy of 5-fluorouracil, being a potential approach for cancer therapy [43].

While morin has shown promising results, it shares some of the limitations of other flavonoids, including poor aqueous solubility, low chemical stability, low bioavailability and rapid excretion. These characteristics restrict its full potential for use in the biomedical field [21,44]. To address these limitations, nanocarriers can be employed to enhance the stability, solubility, and bioavailability of morin for clinical applications [45,46,47]. A range of nano-delivery systems have been developed to improve the anticancer activity of flavonoids and other drugs, including liposomes, micelles, solid-lipid- and polymer-based nanoparticles, as well inorganic nanomaterials (metal- and silica-based nanoparticles and quantum dots) [4,7,48,49,50,51,52].

Mesoporous silica nanoparticles (MSNs) are one of several delivery systems that have been used incorporate various bioactive compounds, including flavonoids [45]. MSNs have demonstrated good biocompatibility and biodegradability, making them a suitable nanocarrier for delivering these compounds [53,54,55,56,57,58]. Several silica-nanoparticle-based formulations are currently undergoing phase I and phase II clinical trials [59,60]. In a clinical trial involving healthy humans, the oral administration of MSNs improved the bioavailability of fenofibrate, a poorly soluble drug [61]. Furthermore, the toxicity of MSNs was investigated in mice following intravenous administration. The results indicated the low toxicity of MSNs at a single dose and repeated administrations [62] and no tissue toxicity after one month in vivo [63]. MSNs have a porous structure with tunable pore sizes and volumes and high surface areas, which is important because these features increase the drug-loading capacity. Furthermore, the surface can readily be functionalized to attach various ligands and consequently enhance the affinity with the tumor microenvironment [21,58,64,65]. Recent studies have reported the success of loading morin in different delivery systems and their therapeutic efficacy against various cancer cell lines [66,67,68,69,70]. However, the effect of morin-loaded MSNs on melanoma therapy remains largely unexplored. The aim of this work was to prepare morin-loaded MSNs (MH-MSNs) and study their potential in the treatment of human melanoma cancer. For this, MSNs were synthesized and loaded with morin hydrate (MH) and subsequently characterized. The morin release from the MSNs in in vitro conditions was investigated and analyzed through several kinetic models. Finally, we evaluated the effects of morin hydrate and morin-loaded MSNs on the cytotoxicity of three melanoma cell lines with different levels of pigmentation: A375, MNT-1 and SK-MEL-28.

## 2. Results and Discussion

### 2.1. Characterization of the Morin Loaded-MSN

**Figure 2:**
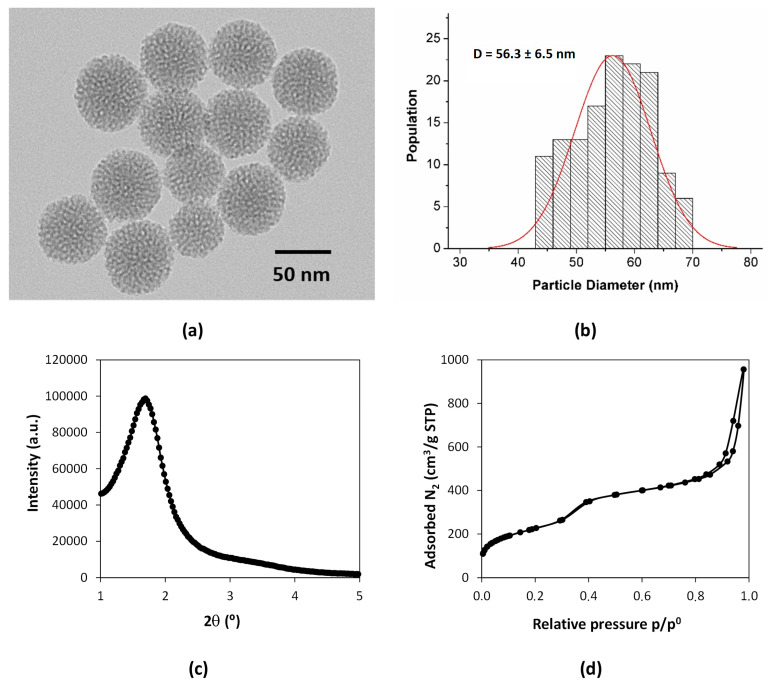
MSN physicochemical characterization. (**a**) TEM micrograph; (**b**) particle size distribution histogram (N = 135 particles); (**c**) low-angle XRD diffraction pattern; (**d**) N_2_ adsorption–desorption isotherms at 77 K.

Mesoporous silica nanoparticles (MSNs) were firstly synthesized and then loaded with morin hydrate (MH). The MSNs showed spheroidal shapes with a mean size of 56.3 ± 6.5 nm determined via TEM analysis (Figure 2a,b). The low-angle powder XRD pattern (Figure 2c) only showed one broad peak at small angles (1.6°), which suggests a short-range ordering porous structure [71]. The textural properties of the MSNs were determined by measuring N_2_ adsorption–desorption. The specific surface area estimated via BET modeling was 816 m^2^/g. The BJH average pore size was 6.8 nm, and the porosity was 1.64 cm^3^/g. The N_2_ adsorption–desorption isotherm (77 K) in Figure 2d showed a steep adsorption step at relative pressure around 0.35 and corresponded to a type IV isotherm (IUPAC classification), being typical of mesoporous materials [72]. At high relative pressure (>0.8), the hysteresis loop was ascribed to interparticle porosity among the dried MSN agglomerates [73].

**Figure 3:**
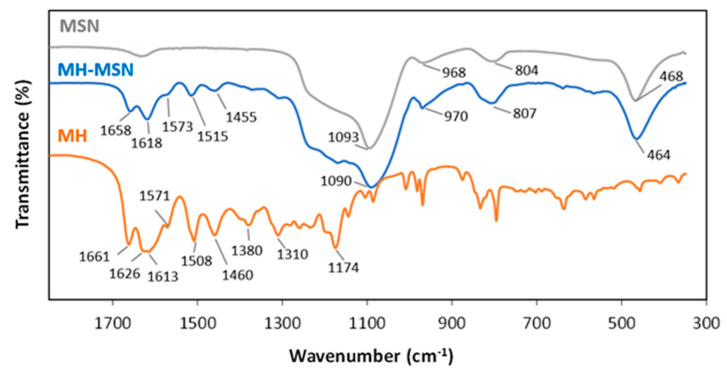
FTIR spectra (1850 to 350 cm^−1^) of MSNs, morin hydrate (MH) and loaded particles (MH-MSNs).

The FTIR spectrum (1850 to 350 cm^−1^) of the synthesized MSNs is depicted in Figure 3 (complete spectrum, 4000–350 cm^−1^, in Figure S3, Supplementary Materials) and showed the typical bands of SiO_2_, namely at 1093 cm^−1^ and 804 cm^−1^ (asymmetric and symmetric Si-O-Si stretching, respectively), 968 cm^−1^ (Si-OH stretching), 468 cm^−1^ (O-Si-O- bending) and 3445 cm^−1^ (-OH stretching) (Table 1) [57,66,74]. The successful removal of the CTAB template from the final MSN is supported by the absence of the characteristic bands of CTAB at 2925 cm^−1^ and 2850 cm^−1^. In contrast, these bands were visible in the FTIR spectrum of the particles prior to calcination (MSN-CTAB, as shown in Figure S3 of the Supplementary Materials). The FTIR spectrum of MH presented broad bands with maximum values at 3376 cm^−1^ and 3154 cm^−1^, ascribed to -OH stretching, with the possible contribution of H-bonding, which is in agreement with the polyhydroxylated structure of MH [75]. Other infrared characteristic bands of MH appeared at 1661 cm^−1^ (-C=O stretching vibration); 1626 cm^−1^ (C=C stretching in aromatic ring); 1613, 1571, 1508 and 1460 cm^−1^ (C=C and C-C stretching); and 1380 and 1310 cm^−1^ (C-OH bending) [67,75]. These MH diagnosis bands appeared in the spectrum of the MH-MSN particles, with slight shifts (Table 1), confirming the successful loading of MH onto MSNs. After MH loading, the carbonyl stretching vibration shifted to lower energies, from 1661 cm^−1^ to 1658 cm^−1^, which indicated an increase in the H-bonding of -C=O morin groups [67], possibly due to the interaction with MSN silanol groups.

**Figure 4:**
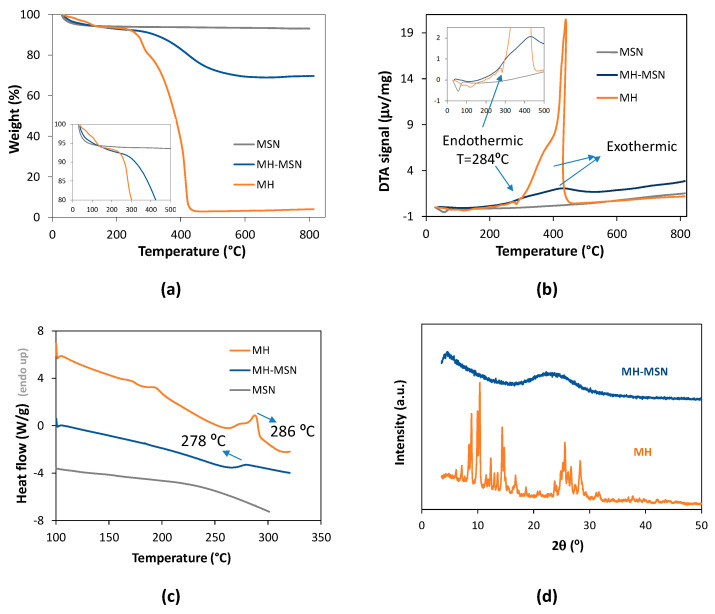
Characterization of MH, MSNs and MH-MSNs: (**a**) TGA; (**b**) DTA; (**c**) DSC and (**d**) XRD.

The MH was loaded onto the MSNs using the evaporation method. The loading procedure was selected based on a previous study that clearly revealed that the evaporation method was more efficient, in comparison with the conventional immersion method, for loading resveratrol, a non-flavonoid polyphenolic compound, onto the MSNs (resulting in a higher loading capacity) [57]. The carbon content increased from 0.04 wt.% in MSNs to 15.99 wt.% in MH-MSNs (Table S1, Supporting Materials). The loading capacity was estimated from the carbon content increase and was found to be 28.3 wt.%. The loading efficiency, calculated using Equation (1), was 99.1%. The thermogravimetric analysis (Figure 4a) shows that loaded particles (MH-MSNs) suffered a significant weight decrease at temperatures higher than 200 °C due to the decomposition of loaded MH. The loading capacity determined via TGA was 27.8%, which is in line with the value estimated from carbon content. The loading efficiency, calculated using Equation (1), was 97.2%. The loading capacity of MH-MSNs was markedly higher than the value achieved in other inorganic [70] or organic [76] nanocarriers. For example, the morin-loading capacity of ceria (CeO_2_) nanoparticles was 10% [70], while in hyaluronic-acid-poly(butyl cyanoacrylate)-based nanoparticles, it was 4.67% [76]. In both studies, the nanoparticles were loaded using the immersion method.

(1) $$Loading\;efficiency\;\left(\%\right)=\frac{m_{loaded\;MH}}{m_{initial\;MH}}\times 100$$

Loaded carriers (MH-MSNs) were further characterized via DTA (differential thermal analysis), DSC and XRD. The DTA curve of bulk MH (Figure 4b) showed an intense exothermic peak with a maximum value at 435 °C, which was due to an oxidative reaction associated with MH decomposition, also visible in MH-MSNs, with less intensity. The DTA curve of bulk MH also showed a marked endothermic peak at 284 °C which was ascribed to the melting transition of crystalline MH. This peak was absent in the curve of MH-MSNs, suggesting that MH changes from a crystalline to an amorphous state in loaded particles. DSC analysis more clearly showed the effect of the encapsulation on the MH melting transition. A marked endothermic peak near 286 °C was observed in the bulk MH curve due to the melting transition (Figure 4c). In MH-MSNs, a much smaller endothermic peak was observed at ca. 278 °C, indicating that only part of MH in loaded particles was crystalline. The decrease in the melting temperature was a consequence of the pore nanoconfinement effect on the MH crystallite size [77,78]. The XRD results (Figure 4d) further corroborated that much of the MH changed from a crystalline to an amorphous state in loaded particles as no diffraction peaks were found in the MH-MSN diffractogram, while the diffractogram of as-purchased MH showed characteristic intense diffractions at 10.2°, 14.2° and 25.4°, among others which were less intense. In contrast, the XRD of bulk MH after being subjected to evaporation in the rotator evaporator (in the conditions used for drug loading but in the absence of MSNs) shows that MH crystallized, although the resulting XRD pattern was distinct from the pattern of the purchased MH (Figure S4, Supporting Materials). Thus, we can conclude that the amorphization of MH is a consequence of the MH loading into the MSNs.

Converting crystalline MH into amorphous state is expected to increase its solubility. This effect has been observed previously for morin encapsulated in polymeric carriers [47,79], as well as for other polyphenols loaded in mesoporous silica nanoparticles [57]. Overall, hydrophobic compounds in the crystalline form are less soluble than their other forms [80].

The surface charge of both synthesized MSN particles and those loaded with MH particles was evaluated through zeta potential measurements at pH 5.2 and 7.4. In both cases, the particles exhibited a negative surface charge at the tested pH levels. Specifically, at physiological pH (pH 7.4), the surface charge decreased from −24.2 mV to −29.1 mV after loading with MH. At the acid pH environment of 5.2, the surface charge of the MSNs was less negative, as expected (−12.2 mV), and showed no marked variation after MH loading (−12.7 mV). Based on the FTIR spectra, it appeared that MH molecules could interact with silanol groups present on the surface of the MSNs. It has been previously reported that the pKa of morin in the acid range is 5.2 [81], which implies that at pH values above 5.2, such as pH 7.4, MH species become anionic as some of the hydroxyl groups undergo deprotonation [66]. Our hypothesis is that the interaction of these anionic MH species with the surface of the MSN causes a reduction in the surface charge, which is consistent with the observed variation in zeta potential at pH 7.4.

### 2.2. In Vitro Release Studies

**Figure 5:**
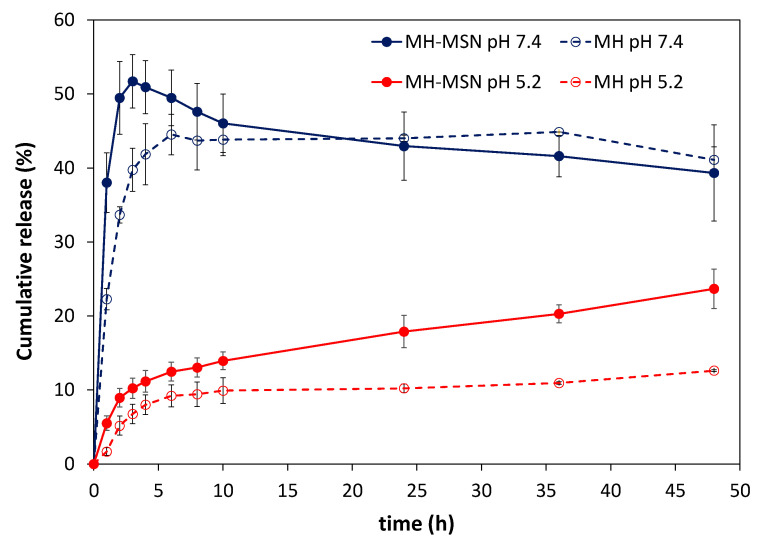
Cumulative release of morin from the non-encapsulated (MH) and encapsulated sample (MH-MSNs) at pH 7.4 and pH 5.2.

Figure 5 shows the cumulative release of MH at pH 5.2 (acid environment in tumorous tissues) and pH 7.4 (physiological pH) at 37 °C. The release of MH was found to be pH-dependent, with a slower release in both encapsulated and bulk forms under acidic conditions. For example, after 24 h, the release from bulk MH was 44.0% at pH 7.4 and decreased to 10.2% at pH 5.2. To conduct the release tests, 500 μg of MH (free or loaded in MSN) was used in a total release medium of 40 mL (accounting partial renewal of the surrounding fluid). The ratio of MH mass to the volume of release medium was 12.5 μg/mL, which corresponded to nearly 8% and 25% of the MH solubility in PBS at pH 7.4 and at pH 5, respectively. These solubilities were measured at 25 °C by Li et al. [44]. It is expected that at 37 °C, the morin solubility will increase up to 5-fold [82], indicating that the ratio of MH mass to the volume of release medium meets sink conditions (defined as the volume of medium at least three times that required to form a saturated solution of a drug). Therefore, the release of MH should not be limited by its solubility value. However, the solubility describes an equilibrium state, whereas drug dissolution is a dynamic process that is characterized by rate, i.e., the amount of drug dissolved per time unit. The release results indicated a greater dissolution rate of MH as pH increased, which is consistent with the increase in MH solubility with increasing pH [83].

At pH 5.2, there was an enhancement in morin release from MH-MSNs compared to non-encapsulated MH. This enhancement resulted in an increase in release from 10.2% to 17.9% after 24 h and from 12.6% to 23.7% after 48 h. The increase in morin release was ascribed to an improvement in the solubility and dissolution rate of amorphous morin compared to the crystalline form [47,79]. At pH 7.4, a short-term increase in morin release was also observed with encapsulation. For example, the release increased from 39.7% to 51.7% after 3 h. However, while at pH 5.2, the morin release from MH-MSN was gradual in time, at pH 7.4, we could observe a distinct profile consisting of a peak release after 3 h followed by a gradual decrease to 39% after 48 h. A careful analysis of the UV-VIS spectra of the release medium (Figure S5, Supporting Materials) shows that at pH 7.4, a new absorption band was observed at ca. 325 nm for both MH and MH-MSN samples, with intensity increasing with time. The appearance of this band indicates the degradation of some released MH molecules via oxidation [83]. At pH 5.2, this band was not observed. This trend agrees with previous studies reporting that MH is less prone to degradation in acidic conditions [83]. It was also reported that light affects the stability of MH more than other conditions, such as temperature. It should be emphasized that the in vitro release studies were performed at 37 °C in dark conditions. The stability of MH in PBS (pH 7.4 and 5.2) in identical conditions was investigated (Figure S6, Supplementary Materials). At pH 7.4, it was noticed that morin degraded 3.8%, 10.4% 12.9% and 15.9% after 3 h, 12 h, 24 h and 48 h, respectively. In contrast, at pH 5.2, the morin degraded < 1% after 12 h and 3% after 48 h.

To investigate the morin release kinetics and understand the release mechanism, the cumulative release data were fitted into various kinetic models: the Korsmeyer–Peppas model (K-P), the Weibull model and a model based on the Noyes–Whitney equation and applying Fick’s law (NWF) [84]. The model fitting was performed for data collected at pH 5.2. At pH 7.4, the exact amount of morin released could not be determined due to the simultaneous degradation of morin. Non-linear regression was performed using the least squares method and the tool solver of the Excel software. The goodness of the fit was assessed based on the analysis of the parameters’ coefficient of determination (R^2^), chi-square (χ^2^) and average relative error (ARE). Respective equations are listed in Table S2 (Supplementary Materials). The Korsmeyer–Peppas (K-P) and the Weibull models showed the best curve fit, with identical values of R^2^, χ^2^ and ARE, as shown in Table 2 and Figure S7 (Supplementary Materials).

The Korsmeyer–Peppas (K-P) model is a semi-empirical model that correlates the release with the time through an exponential function [84,85]. The exponent n in the K-P model is indicative of the transport mechanism. The fitted model shows n = 0.311, which is ≤0.43, and therefore indicates the morin release follows a Fickian diffusion mechanism (also termed as Case I diffusion). The Weibull model also fitted well to the experimental data. This empirical model has been used to successfully describe drug release profiles, including MH release from niosomes [47]. However, due to its empirical nature, the model does not provide insights into the mechanism of release.

### 2.3. Effect of Morin Hydrate and Morin-Loaded MSNs on Cell Viability of Melanoma Cells

**Figure 6:**
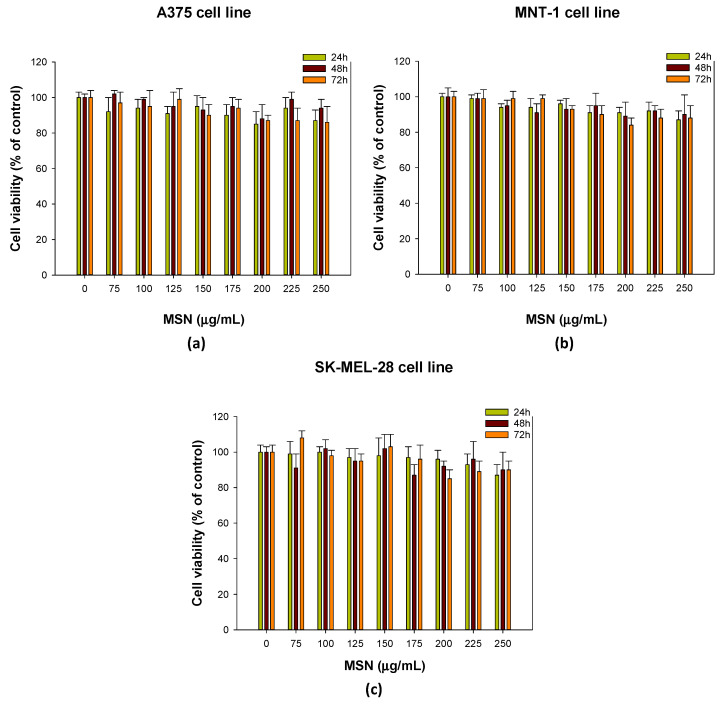
Effect of MSNs (0–250 µg/mL) on cell viability of (**a**) A375 cell line; (**b**) MNT-1 cell line; and (**c**) SK-MEL-28 cell line. Cell viability was evaluated using MTT assay after 24, 48 and 72 h of exposure. Results are presented as mean ± standard deviation (SD).

The cell lines A375 [23,54,86,87], MNT-1 [57,88] and SK-MEL-28 [89,90] have been extensively utilized as melanoma models in various studies. The A375 and SK-MEL-28 cell lines are non-pigmented (amelanotic), in contrast with MNT-1, which is highly pigmented [91]. The cytotoxic effects of morin hydrate (non-encapsulated, MH), MSNs (blank nanoparticles) and morin-loaded MSNs (MH-MSNs) against melanoma cell lines were evaluated using the MTT assay. The MTT assay is one of the most used colorimetric assays and consists of reducing a tetrazolium compound (MTT) into formazan [92]. Colorimetric assays are widely used to assess cellular metabolic activity, being an indicator of toxicity [92,93]. The cell viability of melanoma cell lines was assessed 24, 48 and 72 h after treatment with MH, MSNs, and MH-MSNs. Exposure to MSNs up to 250 µg/mL (Figure 6) did not affect the cell viability of any of the tested cell lines. Even at higher concentrations, MSNs exhibited no cytotoxicity, suggesting that the nanoparticles used in this study show good biocompatibility. Our results are in accordance with several previous works that investigated the cytotoxic effect and interaction of MSNs with melanoma cells [57,94,95].

**Figure 7:**
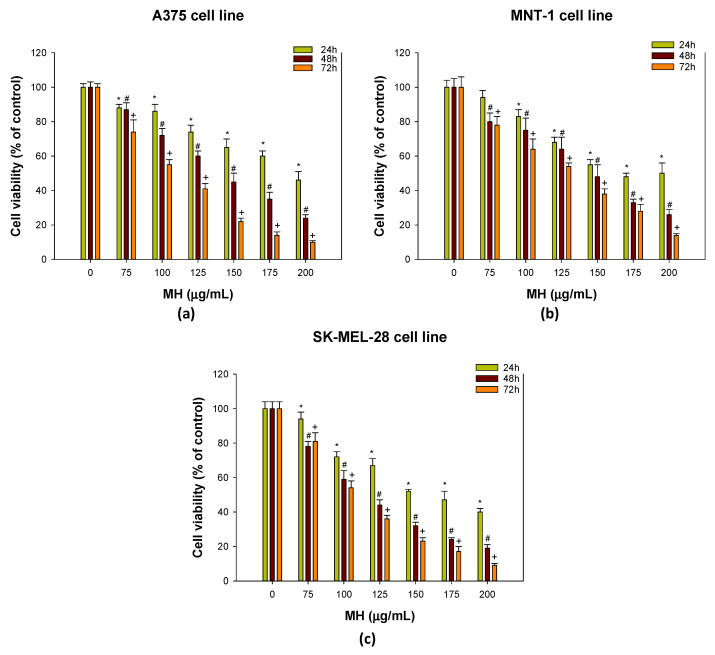
Effect of morin hydrate (MH) (0–200 µg/mL) on cell viability of (**a**) A375 cell line; (**b**) MNT-1 cell line; and (**c**) SK-MEL-28 cell lines. Cell viability was evaluated using MTT assay after 24, 48, and 72 h of exposure. Results are presented as mean ± standard deviation (SD). Values presented are the means ± SD. *, # and + indicate significant differences between control at *p* < 0.05 for 24 h, 48 h and 72 h, respectively.

Figure 7 shows that A375, MNT-1 and SK-MEL-28 cells are sensitive to practically all concentrations of MH tested after 24, 48 and 72 h of exposure. The MH was pre-dissolved in DMSO and culture medium to ensure complete dissolution. The concentration of DMSO varied between 0.22% (lowest MH concentration) and 0.58% (highest MH concentration) in the concentrations tested. Typically, DMSO has cytotoxic effects starting at 1% concentration, depending on the cell type. The effect of DMSO in a concentration range of 0.22–0.58% in the three melanoma cell lines was evaluated, and it was found that it does not have a statistically significant impact on cell viability (Figure S8, Supplementary Materials). The results are in line with those of previous studies with different cell lines [96,97,98]. Furthermore, a study reported that the flavonoid quercetin was able to reverse the effect of DMSO, and it was actually quercetin that decreased the cell viability of human lens epithelial cells [99].

The cell viability of A375 (Figure 7a) and SK-MEL-28 cell lines (Figure 7c) decreased significantly at all exposure times for all concentrations of MH tested. In the case of the MNT-1 cell line (Figure 7b), the cell viability also decreased in a similar pattern, except for the exposure of 24 h at 75 µg/mL, which did not induce significant differences on cell viability. Moreover, the dependence of cytotoxicity on MH concentration and time was also evident, with the highest concentration after 72 h of exposure leading to less than 20% of live cells. Furthermore, we conducted tests to evaluate the effects of MH on the three cell lines using the same cell density (20,000 cells/mL) for 24 h, 48 h and 72 h of exposure. This approach aimed to gain a better understanding of the results and facilitate comparisons. A similar effect of MH was observed, with all cell lines exhibiting a consistent decline in cell viability as the concentration of MH increased (Figure S9, Supplementary Materials).

The time-dependent toxicity of MH is also evidenced in Table 3, which shows that the half maximal inhibitory concentration (IC_50_) for MH decreased with increased time exposure. The MH concentrations that inhibited 50% of the growth of A375 were 194.4, 140.3 and 105.0 µg/mL at 24, 48 and 72 h, respectively. The IC_50_ for MNT-1 for 24 h of exposure was 178.0 µg/mL, less than the A375 cell line, but for 48 and 72 h, it was 142.0 and 124.4 µg/mL, respectively, slightly higher when compared to A375 cells. Regarding SK-MEL-28, the IC_50_ values were 163.1, 115.1 and 107.4 µg/mL for 24, 48, and 72 h, respectively. Although there were not many differences, at the shortest exposure period (24 h), SK-MEL-28 was the most sensitive cell line, but at 72 h of exposure, A375 was revealed to be the most sensitive for MH.

Previous studies using different cell lines have revealed the anticancer activity of flavonoids such as fisetin [24,100], apigenin [101], genistein [102,103], quercetin [104] and luteolin [26]. There is, however, scarce information on the potential of morin in melanoma treatment, which underscores the significance of this study. Nevertheless, the limited existing research is consistent with our findings. For instance, Lee et al. [32] reported that morin at 200 µM (60 µg/mL) reduced the SK-MEL-2 cells’ viability to 75%, mainly by inducing cell apoptosis and ROS production. The cell viability of the CD133^+^ melanoma MV3 cell subpopulation was significantly reduced by morin, and the in vivo study also showed the suppression of melanoma tumor growth [33]. Furthermore, Li et al. [38] used a morin derivative that induced a decrease in cell proliferation and promoted apoptosis in B16-F10 melanoma cells. Morin was also effective in reducing cell viability in a breast cancer cell line, MDA-MB-231, particularly at higher concentrations (100 and 200 µM), and significantly inhibited colony formation capacity [105,106]. Morin also decreased cell viability in human colon cancer cell line HCT-116 (morin concentration ≥ 250 µM) [41] and in SW480 cells (morin concentration ≥ 100 µM) [107]. Comparing our findings with those of various studies highlights the heterogeneity of cancer and emphasizes the importance of exploring new therapies.

**Figure 8:**
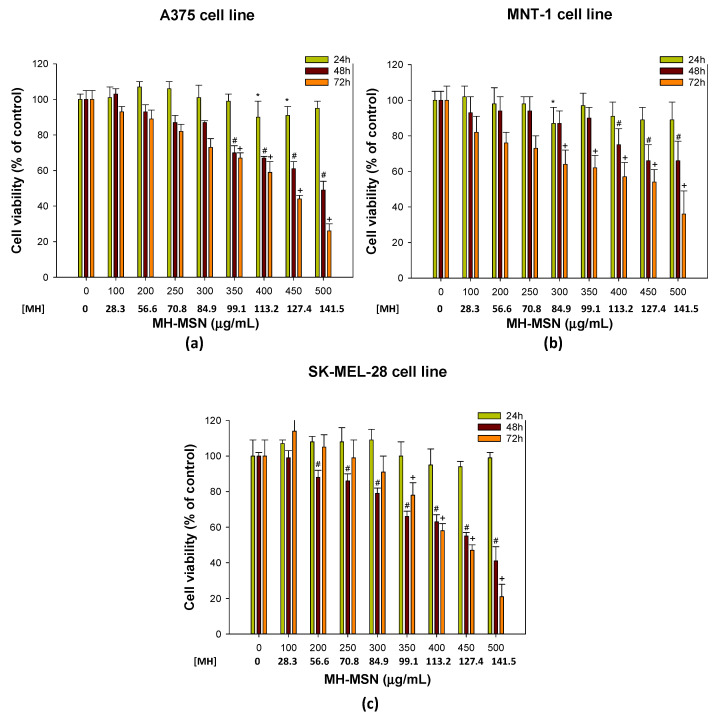
Effect of morin-loaded MSNs (MH-MSNs) (0–500 µg/mL) on cell viability of (**a**) A375 cell line; (**b**) MNT-1 cell line; and (**c**) SK-MEL-28 cell line. Cell viability was evaluated using MTT assay after 24, 48 and 72 h of exposure. [MH] represents the estimated concentration of MH loaded in the nanosystem. Results are presented as mean ± standard deviation (SD). *, # and + indicate significant differences between control at *p* < 0.05 for 24 h, 48 h and 72 h, respectively.

The morin-loaded MSNs were then tested in the three melanoma cell lines. Exposure for 24 h with morin-loaded MSNs (Figure 8) did not induce significant differences in the melanoma cells tested, and the IC_50_ for 24 h was markedly above the range of the MH-MSN concentrations tested. The MH-MSNs at 507.7 and 414.0 µg/mL reduced the cell viability by 50% in A375 cells after 48 and 72 h of exposure, respectively (Table 3). MH-MSNs at concentrations of 582.4 and 468.3 µg/mL significantly inhibited MNT-1 growth by 50% after 48 and 72 h, respectively. Regarding SK-MEL-28, the half maximal inhibitory concentration (IC_50_) was achieved at 464.1 µg/mL for 48 h and 423.3 µg/mL for 72 h. The cytotoxicity of MH-MSNs increased with concentration, especially for 48 and 72 h, which agrees with our observations when using MH. Notably, a significant decrease in the cell viability of A375, MNT-1 and SK-MEL-28 cells was noticed between MH-MSN concentrations of 350 and 400 µg/mL after 48 and 72 h of exposure. We observed a significant decrease in the viability of A375, MNT-1, and SK-MEL-28 cells when exposed to MH-MSN concentrations between 350 and 400 µg/mL after 48 and 72 h. While the differences were minor, it appears that the A375 and SK-MEL-28 cell lines, which are amelanotic (i.e., non-pigmented), were slightly more sensitive than the highly pigmented MNT-1 cells, particularly with prolonged exposure to both MH and MH-MSN treatments.

Considering the loading capacity of 28.3% determined in Section 3.1, the concentration of MH entrapped in the MH-MSNs at the IC_50_ values for 48 and 72 h was estimated. For the A375 cell line, the entrapped MH concentration was 143.7 and 117.2 µg/mL for 48 and 72 h, respectively. Regarding MNT-1, it was 164.8 and 132.5 µg/mL for 48 and 72 h, respectively. Finally, the estimated MH concentration in the MH-MSNs for the SK-MEL-28 cell line was 131.3 and 119.8 µg/mL for 48 and 72 h, respectively. The MH concentrations that reduced the cell viability by 50% presented in Table 3 for bulk MH (pre-dissolved in DMSO to ensure total dissolution) were very similar to the estimated MH concentration entrapped in the MSNs. These results agree with the similar release values of both MH and MH-MSN at pH 7.4 for longer exposure periods.

Previous studies have shown that morin encapsulation significantly enhanced its anticancer properties by improving its intracellular delivery when compared to free morin, inducing cell death in different cancer cell lines, such as lung [79,108,109], cervical [46] and breast cancer [70,110]. However, there is a limited amount of research on the use of mesoporous silica nanoparticles loaded with morin for melanoma treatment. Recently, it was reported that modified mesoporous silica nanoparticles (SBA-16) loaded with morin exhibited cytotoxic effects towards the cell lines HL-60 and U-266, which are acute promyelocytic leukemia cells and multiple myeloma cells, respectively [67]. These results are in agreement with our findings. In addition, a study using liposomes demonstrated that the anticancer effect of morin against different cancer cell lines was enhanced after encapsulation when compared to free morin [111]. Ghosh and co-workers used morin-loaded human serum albumin nanoparticles against the breast cancer cell line MDA-MB-468. The protein nanoparticles increased the solubility of morin, leading to more potent cancer cell destruction than morin alone [112,113]. These studies support the concept that the encapsulation of flavonoids such as morin enhances their release and consequently induces cancer cell death.

## 3. Materials and Methods

### 3.1. Preparation of Morin-Loaded MSNs

#### 3.1.1. Reagents

All chemicals were of analytical grade and utilized as received. Cetyltrimethylammonium bromide (CTAB; 99%), tetraethoxysilane (TEOS; 99%), morin hydrate (MH, >85%), phosphate-buffered saline (PBS) pH 7.4 (0.01 M; with NaCl 138 mM and KCl 2.7 mM) and cylindrical dialysis cellulose membranes (3.5 kDa MWCO) were acquired from Sigma-Aldrich (St. Louis, MO, USA). Triethanolamine (TEA; 99%) and ethanol (CH_3_CH_2_OH; >99%) were acquired from Fisher Scientific (Hampton, NH, USA). Hydrochloric acid (HCl; 37%) was bought from VWR International (Radnor, PA, USA). Deionized water obtained with a Milli-Q system comprising a 0.22 μm filter (Synergy Equipment, Millipore, Burlington, MA, USA) was employed in all experiments.

#### 3.1.2. Mesoporous Silica Nanoparticles’ (MSNs’) Preparation

The MSNs were synthesized using a simple method adapted from the literature with some adjustments [114]. Succinctly, 0.5 g of surfactant (CTAB) was dissolved in 20 mL of Milli-Q water. The solution was stirred (450 rpm) in a round-bottom flask immersed in an oil bath. When the solution reached 95 °C, 100 µL of TEA was added and stirred for another 30 min. Then, 1.5 mL of TEOS was added dropwise, and the solution was allowed to react for 2 h. All synthesis procedures were performed with temperature control and in reflux conditions to guarantee that concentrations were preserved. To eliminate the remaining reactants, MSNs were centrifuged at 13,000 rpm for 10 min before being cleaned three times with ethanol. To remove the CTAB template, MSNs were extracted for 6 h using a solution of HCl (1% *v*/*v*) in ethanol under reflux conditions. The remaining HCl was cleaned out with one final ethanol wash. Lastly, MSNs were calcinated in a muffle furnace (Termolab, Águeda, Portugal) for 5 h at 550 °C to completely eliminate the surfactant. After all experimental steps, the yield of MSN production was 37%.

#### 3.1.3. Loading of Morin to MSNs

Morin was loaded via the rotary evaporation technique. Briefly, 40 mg of morin hydrate (MH) was dissolved in a round-bottom flask with 10 mL of ethanol and placed in the ultrasonic bath for 2 min. After that, 100 mg of MSN was added to the flask and dispersed in the ultrasonic bath for 5 min. To obtain the morin-loaded MSNs, the solvent was then gradually evaporated in a rotary evaporator at 50 °C until all ethanol was removed. The final solids (MH-MSNs) were scraped from the flask, kept at room temperature, and covered with aluminum foil to prevent light exposure.

#### 3.1.4. Characterization of the Synthesized and Loaded MSNs

The morphology and size of the MSNs were evaluated via transmission electronic microscopy (TEM) using a Hitachi H-9000 instrument (Hitachi, Tokyo, Japan) operating at 300 kV. Samples were prepared through the immersion of a copper grid coated with an amorphous carbon film in diluted suspensions of the nanoparticles in ethanol. X-ray diffractograms were obtained using a PANalytical Empyrean diffractometer (Malvern PANalytical, Malvern, UK) with Kα(Cu) radiation and a curved graphite monochromator. The analysis in reflection geometry was performed in the range of 3.5° < 2θ < 50° (step 0.026°), while analysis in transmission geometry was performed in the range of 1° < 2θ < 5° (step 0.026°). N_2_ adsorption-desorption isotherms were obtained using a Micrometrics Gemini V2.0 (Micromeritics Instrument Corp., Norcross, GA, USA) system at 77 K. Samples were pre-treated at 473 K for 5 h under N_2_ gas. The pore size and surface area were calculated from desorption branches via the Barrrett–Joyner–Halenda (BJH) method and the Brunauer–Emmett–Teller (BET) isotherm, respectively. Fourier-transform infrared spectroscopy (FTIR) spectra were collected on a Mattson 7000 spectrometer (Mattson Instruments Inc., Madison, WI, USA) using KBr pellets; the measurement parameters were 256 scans per sample in the range of 4000–300 cm^−1^ with a resolution of 2 cm^−1^. The carbon and hydrogen content were ascertained via elemental analysis on a Leco TruSpec-Micro CHNS 630-200-200 (LECO, Saint Joseph, MI, USA). The amount of morin loaded on the MSNs was calculated from carbon content and verified with thermogravimetric analysis (TGA). TGA was performed in a Hitachi STA300 instrument (Hitachi, Tokyo, Japan) heated from 30° to 800 °C at 5 °C/min under a N_2_:O_2_ atmosphere (3:1). The differential thermal analysis (DTA) of the materials was obtained simultaneously in the same equipment and conditions. Heating curves of the materials were obtained using a differential scanning calorimeter (Diamond DSC, Perkin Elmer, Waltham, MA, USA). Accurately weighed samples (2–5 mg) were loaded into a non-hermetically crimped aluminum pan and heated under a nitrogen purge at the rate of 50 mL/min, from 100 to 350 °C at a heating rate of 10 °C/min. Data were analyzed using TA Instruments’ Universal Analysis 2000 software V4.5. UV–VIS spectra were obtained in a SYNERGY HT microplate reader (BioTek Instruments, Winooski, VT, USA). The surface charge of the nanoparticles was assessed using zeta potential measurements, which were performed through electrophoretic light scattering performed using a Zeta Sizer Nano Plus equipment (Malvern Instruments, Harris County, TX, USA) by suspending the nanoparticles at different pH values. The equilibration time established was 120 s (for each sample), and 3 measurements were performed with 100 runs each.

#### 3.1.5. In Vitro Release Studies

The morin release was investigated in PBS at pH 7.4 and pH 5.2. Several suspensions were prepared by dispersing morin-loaded MSNs (equivalent to 500 µg of bulk morin) and MH (for comparison) into 1 mL of PBS. The suspensions were then placed in a dialysis membrane (3.5 kDa cut-off) and immersed in 29 mL of PBS. The resulting solutions were gently stirred at 37 °C while being protected from light exposure. After specific periods of time, 1 mL aliquots of solution were withdrawn for analysis and immediately replaced with the same volume of PBS. These aliquots were used to monitor the amount of morin released during 48 h.

The aliquots were analyzed with a UV–VIS spectrophotometer (BioTeK, SYNERGY HT microplate reader). The concentration of morin in the aliquots was estimated according to the Lambert–Beer law, which correlates the absorbance with the concentration of the sample, using the calibration curves that were prepared for the morin solutions at pH 7.4 and 5.2. Several morin solutions of known concentrations were prepared, and their absorbance at λ = 390 nm for pH 7.4 and λ = 384 nm for pH 5.2 was measured. The absorbance vs. concentration was plotted (Figures S1 and S2, Supplementary Materials) and followed a linear correlation (r^2^ = 0.9987 and r^2^ = 0.9993 for pH 7.4 and 5.2, respectively). The cumulative release of morin, in a percentage, was given via Equation (S1) (Supplementary Materials). The release curves presented are an average of triplicates.

#### 3.1.6. Morin Stability Studies

The stability of morin in PBS was evaluated by following its concentration using UV-VIS spectroscopy. The solutions were prepared by dissolving MH in 50 mL of PBS (pH 5.2 and 7.4) and maintaining it at 37 °C under constant stirring while being protected from light. Aliquots were withdrawn for analysis at the following time intervals: 0 h, 3 h, 6 h, 12 h, 24 h, 36 h and 48 h.

### 3.2. In Vitro Cell Studies

#### 3.2.1. Reagents

3-(4,5 dimethyl-2-thiazolyl)-2,5-diphenyl tetrazolium bromide (MTT; 98%) and dimethyl sulfoxide (DMSO; ≥99.7%) were acquired from Sigma-Aldrich (St. Louis, MO, USA). Dulbecco’s Modified Eagle’s Medium (DMEM), trypsin-ethylendiaminetetraacetic acid EDTA (0.25% trypsin and 1 mM EDTA), fetal bovine serum (FBS), l-glutamine and fungizone (250 U mL^−1^) were obtained from Gibco, Life Technologies (Grand Island, NY, USA). Penicillin–streptomycin (10,000 U mL^−1^) was purchased from Grisp (Porto, Portugal).

#### 3.2.2. In Vitro Cell Culture

A375 and SK-MEL-28 human melanoma cell lines were acquired from the European Collection of Authenticated Cell Cultures (ECACC). The MNT-1 melanoma cell line was kindly supplied by Dr. Manuela Gaspar (iMed.ULisboa, Lisbon, Portugal). Cell lines were cultured at 37 °C under 5% CO_2_ in DMEM culture medium, supplemented with 10% FBS, 2 mM L-glutamine, 1% penicillin–streptomycin (100 U/mL penicillin, 100 μg/mL streptomycin) and 1% fungizone (250 U mL^−1^). Cells were monitored to ensure satisfactory confluence and morphology using an inverted phase-contrast Eclipse TS100 microscope (Nikon, Tokyo, Japan). When 70–80% of confluence was reached, cells were trypsinized with trypsin-EDTA (0.25% trypsin and 1 mM EDTA) and used in the in vitro cell viability assays (Section 3.2.3).

#### 3.2.3. Exposure Treatment and Determination of Cytotoxicity

A morin hydrate stock solution was prepared by dissolving MH in 5% DMSO and culture medium to ensure complete dissolution. The final volume was adjusted, and the final concentration of DMSO at the highest concentration tested was 0.58%. Encapsulated MH was dispersed in culture medium. The three cell lines were seeded in 96-well plates. A375 cells were seeded at 40,000, 25,000 and 20,000 cells/mL for 24, 48 and 72 h of exposure, respectively. MNT-1 cells were seeded at 35,000, 25,000 and 15,000 cells/mL for 24, 48 and 72 h of exposure, respectively, and SK-MEL-28 at 50,000, 20,000 and 10,000 cells/mL for 24, 48 and 72 h of exposure, respectively. After 24 h, the medium was replaced with the exposure solutions containing (i) MH (0, 75, 100, 125, 150, 175 and 200 µg/mL), (ii) MSNs (0.75, 100, 125, 150, 175, 200, 225 and 250 µg/mL), or (iii) MH-MSNs (0, 100, 200, 250, 300, 350, 400, 450 and 500 µg/mL). As a negative control, cells exposed to the control medium were used. Cell viability was measured at 24, 48 and 72 h following exposure.

Cell viability was assessed via the MTT colorimetric assay [115]. At the end of the exposure time, the wells were emptied and replaced with fresh medium (100 µL), 50 µL of MTT (1 mg/mL in PBS) and then incubated for 4 h at 37 °C under a 5% CO_2_ humidified atmosphere. Afterwards, the culture medium containing MTT was withdrawn and changed with 150 µL of DMSO for formazan crystal solubilization. Samples’ absorbance was measured at 570 nm with a BioTek Synergy HT plate reader (Synergy HT Multi-Mode, BioTeK, Winooski, VT, USA).

#### 3.2.4. Statistical Analysis

The results were presented as mean ± standard deviation. SigmaPlot version 14.0 (Systat Software, San Jose, CA, USA) was used for statistical analysis. Data were analyzed via one-way ANOVA (*p* < 0.05) as well as via Dunnett’s test (*p* < 0.05).

## 4. Conclusions

In this study, we prepared mesoporous silica nanoparticles (MSNs) to act as carriers for morin, an anticancer drug. The MSNs were synthesized and morin hydrate (MH) was efficiently loaded onto them using the evaporation technique. The loading capacity was 28.3% and the loading efficiency was 99.1%. The encapsulation of MH facilitated its release in vitro, likely due to the conversion of crystalline MH into an amorphous state upon loading onto MSNs, which increased its solubility and dissolution rate. The enhancement of MH release in acidic pH suggests that morin-loaded MSNs could be a promising nano-delivery system to treat melanoma skin cancer. However, we detected MH degradation at pH 7.4, which is a point that should be improved in future studies. MH and MH-MSNs induced concentration- and time-dependent cytotoxicity in the A375, MNT-1 and SK-MEL-28 melanoma cell lines. Notably, the A375 and SK-MEL-28 cell lines were slightly more sensitive to both the MH and MH-MSN exposure treatments than the MNT-1 cells.

We hope that this study will inspire further in vitro studies, such as using 3D models, and in vivo studies to confirm the efficacy of morin-loaded MSNs.

## Figures and Tables

**Table 1:** Assignment of infrared bands (cm^−1^) for MSNs, morin hydrate (MH) and loaded particles (MH-MSNs) [57,66,67,74,75].

	MSNs	MH	MH-MSNs
ν (-O-H)	3445 (vs)	3376 (vs); 3154 (vs)	3428 (vs)
ν_asym_ (Si-O-Si)	1093 (vs)	--	1090 (vs)
ν_sym_ (Si-O-Si)	804 (m)	--	807 (m)
ν (Si-OH)	968 (m)	--	970 (m)
δ (O-Si-O)	468 (s)	--	464 (s)
ν (C=O)	--	1661 (vs)	1658 (s)
ν (C=C)	--	1626 (vs)	--
ν (C=C;C-C)	--	1613 (vs); 1571 (m); 1508 (vs); 1460 (s)	1618 (s); 1573 (w); 1515 (m); 1455 (m)
δ (C-OH)	--	1310 (m); 1380 (m)	1306 (w); 1369 (w)
δ (C-C-O)	--	1174 (vs)	1168 (s)

vs—very strong; s—strong; m—medium; w—weak; vw—very weak; ν—stretching vibration; δ—bending vibration; asym—asymmetric; sym—symmetric.

**Table 2:** Kinetics parameters and goodness of the fits in morin release from loaded MSN at pH 5.2. k_F_: first-order rate constant independent of drug concentration; k_KP_: constant of K-P model; NWF: Noyes–Whitney and Fick’s law model; K–P: Korsmeyer–Peppas model; ARE: average relative error.

Model		MH-MSN, pH 5.2
NWF	k_F_ (min^−1^)	1.2 × 10^−4^
	R^2^	0.8020
	χ^2^	1.575
	ARE (%)	49.5
K–P	k_KP_ (min^−n^)	0.0192
	n	0.3114
	R^2^	0.9903
	χ^2^	0.0044
	ARE (%)	5.03
Weibull	α (min^−β^)	56.96
	β	0.3380
	R^2^	0.9903
	χ^2^	0.0042
	ARE (%)	5.04

**Table 3:** Half maximal inhibitory concentration (IC_50_) of morin and morin-loaded MSNs to A375, MNT-1 and SK-MEL-28 melanoma cells.

Cell Line		IC_50_ (µg/mL)		
		24 h	48 h	72 h
A375	MH	194.4	140.3	105.0
	MH-MSN	977.5	507.7	414.0
MNT-1	MH	178.0	142.0	124.4
	MH-MSN	1853.1	582.4	468.3
SK-MEL-28	MH	163.1	115.1	107.4
	MH-MSN	818.8	464.1	423.3

## Data Availability

The data reported are contained within the manuscript and Supplementary Material.

## Supplementary Materials

The following supporting information can be downloaded at: https://www.mdpi.com/article/10.3390/molecules28124776/s1, Figure S1: Calibration curve for morin quantification in PBS pH 7.4; Figure S2: Calibration curve for morin quantification in PBS pH 5.2; Figure S3: FTIR spectra (4000 to 350 cm^−1^) of MSNs, morin hydrate (MH) and loaded particles (MH-MSNs); Figure S4: XRD spectra of commercially available morin hydrate (MH) and morin after its dissolution and removal of ethanol using a rotary evaporator (MEv); Figure S5: UV-VIS spectra of the release medium over time during in vitro release tests; Figure S6: Morin concentration (%) over time in PBS 5.2 and 7.4, assessed via UV-VIS spectroscopy analysis; Figure S7: Morin release from MH-MSNs at pH 5.2 and kinetic model fitting; Figure S8: Effect of DMSO on cell viability of A375 cell line, MNT-1 cell line and SK-MEL-28 cell lines. Cell viability was evaluated using MTT assay after 24, 48 and 72 h of exposure. Results are presented as mean ± standard deviation (SD); Figure S9: Effect of morin hydrate (MH) (0–200 µg/mL) on cell viability of (a) A375 cell line; (b) MNT-1 cell line; and (c) SK-MEL-28 cell line. Cell viability was evaluated using MTT assay after 24, 48 and 72 h of exposure, using the same density (20,000 cells/mL). Results are presented as mean ± standard deviation (SD). *, # and + indicate significant differences between control at *p* < 0.05 for 24 h, 48 h and 72 h, respectively; Table S1: Elemental analysis of MSNs, MH and loaded sample MH-MSNs; Table S2: Non-linear equations of the Korsmeyer–Peppas (K-P) model, the Weibull model and an exponential equation based on the Noyes–Whitney equation and applying Fick’s law (NWF) and equations to evaluate the goodness of the fittings.

## Sample Availability

Samples of the compounds MSN and MH-MSN are not available from the authors.
