# Supplementary material for: Morin Hydrate Encapsulation and Release from Mesoporous Silica Nanoparticles for Melanoma Therapy
Source: Molecules. 2023 Jun 15;28(12):4776. doi: 10.3390/molecules28124776 (PMC10302569; doi:10.3390/molecules28124776)
Supplement: Supplementary file 1 [file molecules-28-04776-s001.zip › molecules-2360486-supplementary.pdf]

molecules

Supplementary Materials

Morin Hydrate Encapsulation and Release from Mesoporous Silica Nanoparticles for Melanoma Therapy

Catarina Cunha [1,2,†], Diogo Marinheiro [2,†], Bárbara J. M. L. Ferreira [2], Helena Oliveira [1,*] and Ana L. Daniel-da-Silva [2,*]

[1] Department of Biology, CESAM-Centre for Environmental and Marine Studies, University of Aveiro, 3810-193 Aveiro, Portugal; catarinafilipacunha@ua.pt
[2] Department of Chemistry, CICECO-Aveiro Institute of Materials, University of Aveiro, 3810-193 Aveiro, Portugal; diogomarinheiro@ua.pt (D.M.); barbaraferreira@ua.pt (B.J.M.L.F.)
* Correspondence: holiveira@ua.pt (H.O.); ana.luisa@ua.pt (A.L.D.-d.-S.)
† These authors contributed equally to this work.

Page

A. Calibration curves for morin hydrate quantification………………………………. **2**

B. Characterization of the MSN, MH and MH-MSN………………………………….. **3**

C. *In vitro* release tests…………………………………………………………………... **5**

D. *In vitro* cell studies…………………………………………………….……………... 9

A. Calibration curves for morin hydrate quantification

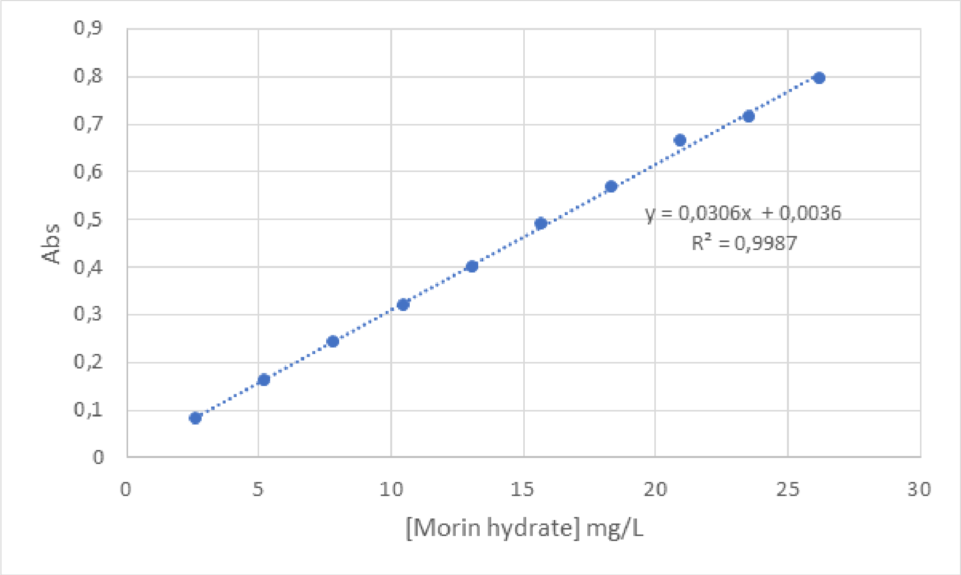

Figure S1. Calibration curve for morin quantification in PBS pH 7.4.

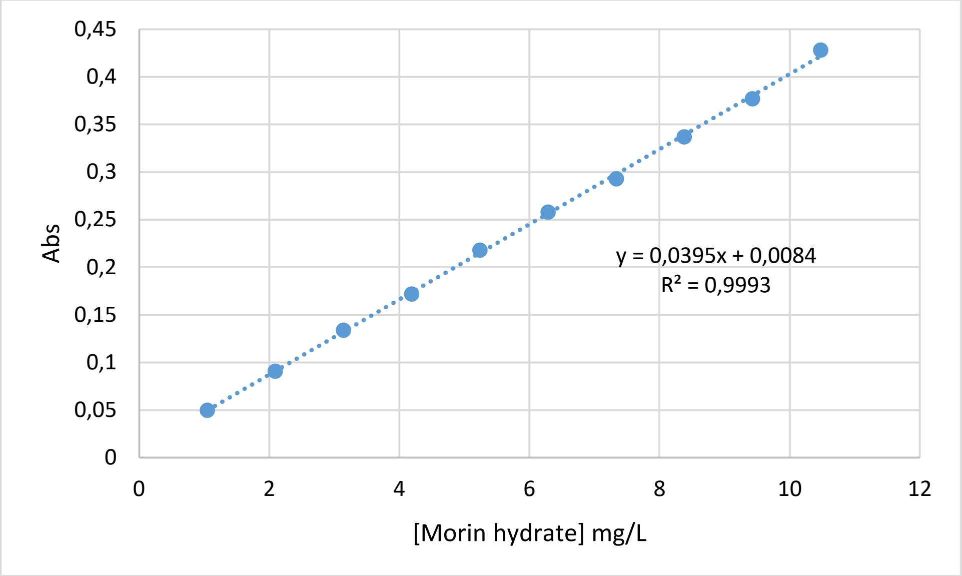

Figure S2. Calibration curve for morin quantification in PBS pH 5.2.

B. Characterization of the MSN, MH and MH-MSN

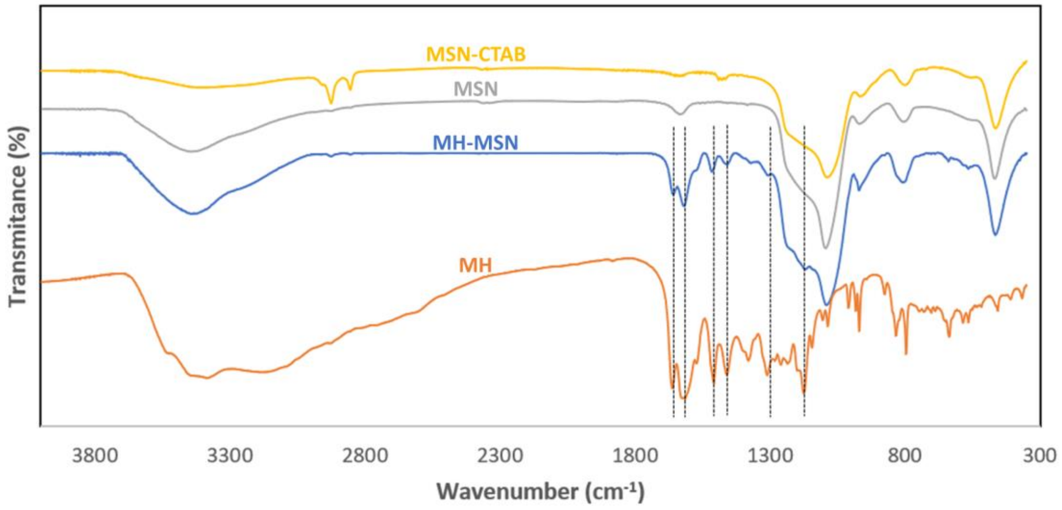

Figure S3. FTIR spectra (4000 to 350 cm^{-1}) of MSN before template removal (MSN-CTAB), MSN, morin hydrate (MH) and loaded particles (MH-MSN).

Table S1. Elemental analysis of MSN, MH and loaded sample MH-MSN.

	MSN	MH	MH-MSN
%C	0.040	56.37	15.99
%H	1.424	4.954	2.183

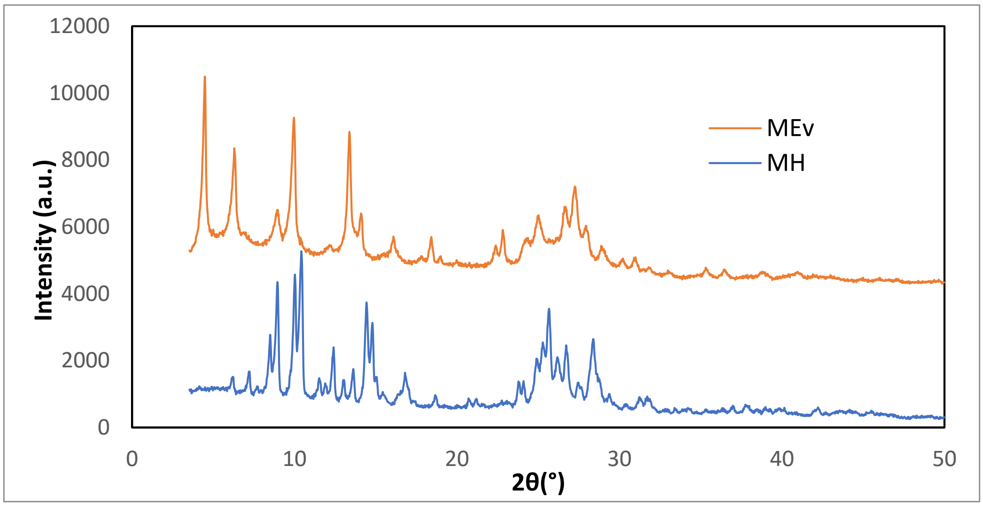

Figure S4. XRD spectra of commercially available morin hydrate (MH) and morin after its dissolution and removal of ethanol using a rotary evaporator (MEv).

C. *In vitro* release tests

The cumulative release percentage of morin was calculated using Equation (S1):

$$\text{Cumulative release (\%)} = \frac{\text{Volume of aliquot withdrawn (mL)}}{\text{Total volume (mL)}} \times P_{(t-1)} + P_t \quad \text{(S1)}$$

Where P_t corresponds to the percentage released at time t and $P_{(t-1)}$ corresponds the percentage released previous to time t. The data presented in the release curves are an average of triplicates.

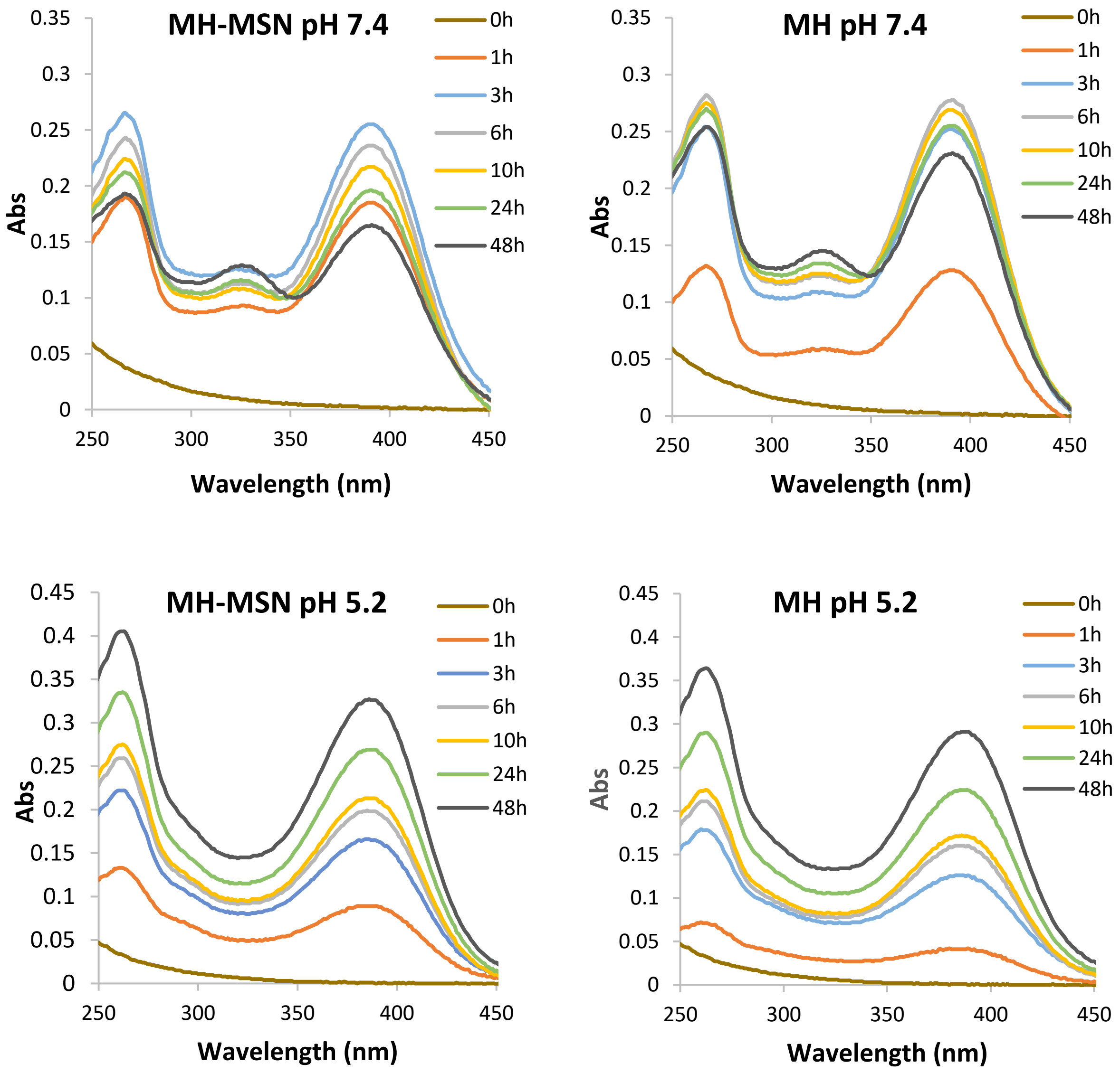

Figure S5. UV-VIS spectra of the release medium over time during *in vitro* release tests.

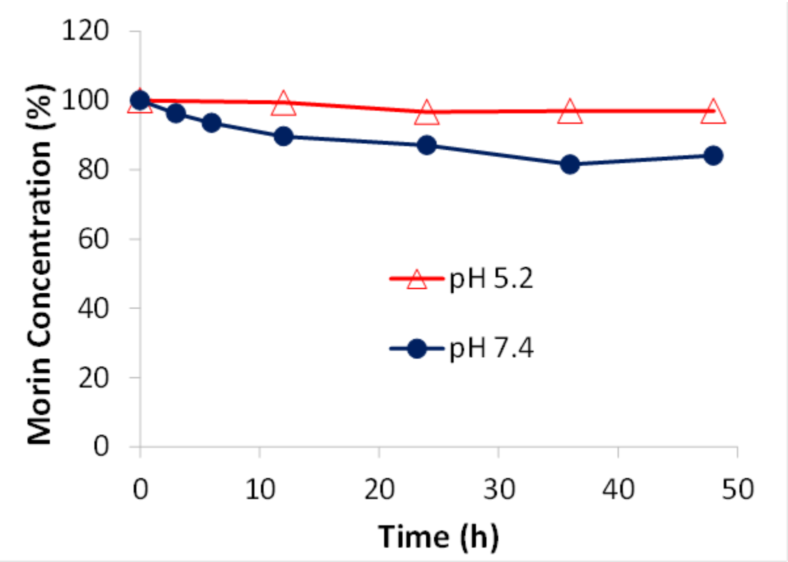

Figure S6. Morin concentration (%) over time in PBS 5.2 and 7.4, assessed by UV-VIS spectroscopy analysis.

Table S2. Non-linear equations of the Weibull model, the Korsmeyer–Peppas model (K-P), and an exponential equation based on the Noyes–Whitney equation and applying Fick's law (NWF) and equations to evaluate the goodness of the fittings.

Kinetic model	Non-linear equation	Model characteristics/parameters
**NWF**	$M_t/M_\infty = 1 - e^{-k_F t}$	M_t/M_∞ - fraction of drug released at time t<br>k_F - first-order rate constant
**K-P**	$M_t/M_\infty = k_{KP}.t^n$	- Semi-empirical model<br>M_t/M_∞ - fraction of drug released at time t<br>k_{KP} - constant incorporating the characteristics of the system<br>n - indicates the transport mechanism; system geometry dependent
**Weibull**	$M_t/M_\infty = 1 - e^{\left(-\frac{(t-T_i)^\beta}{\alpha}\right)}$	- Empirical Model<br>M_t/M_∞ - fraction of drug released at time t<br>α - time process<br>Ti - lag time before the onset of the drug release (in most cases zero)<br>β - shape parameter

Goodness of the fittings	Equation	Parameters
**Coefficient of determination**	$R^2 = 1 - \frac{\sum_{i=1}^{n}(y_i - \hat{y}_i)^2}{\sum_{i=1}^{n}(y_i - \bar{y})^2}$	y_i – experimental value<br>$\hat{y}_i$ – model predicted value<br>$\bar{y}$– mean value of the experimental data<br>n – sample size
**Chi-square**	$\chi^2 = \sum_{i=1}^{n}\frac{(y_i - \hat{y}_i)^2}{\hat{y}_i}$	
**Average Relative Error**	$ARE = \frac{100}{n}\sum_{i=1}^{n}\left\lvert\frac{.\hat{y}_i - y_i}{y_i}\right\rvert$	

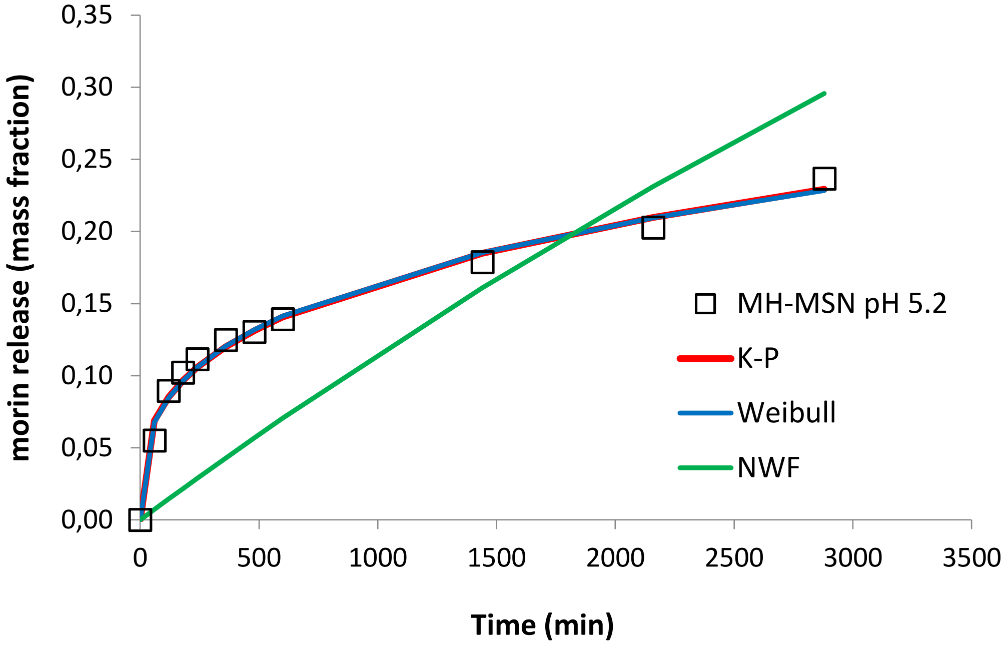

Figure S7. Morin release from MH-MSN at pH 5.2 and kinetic model fitting.

D. *In vitro* cell studies

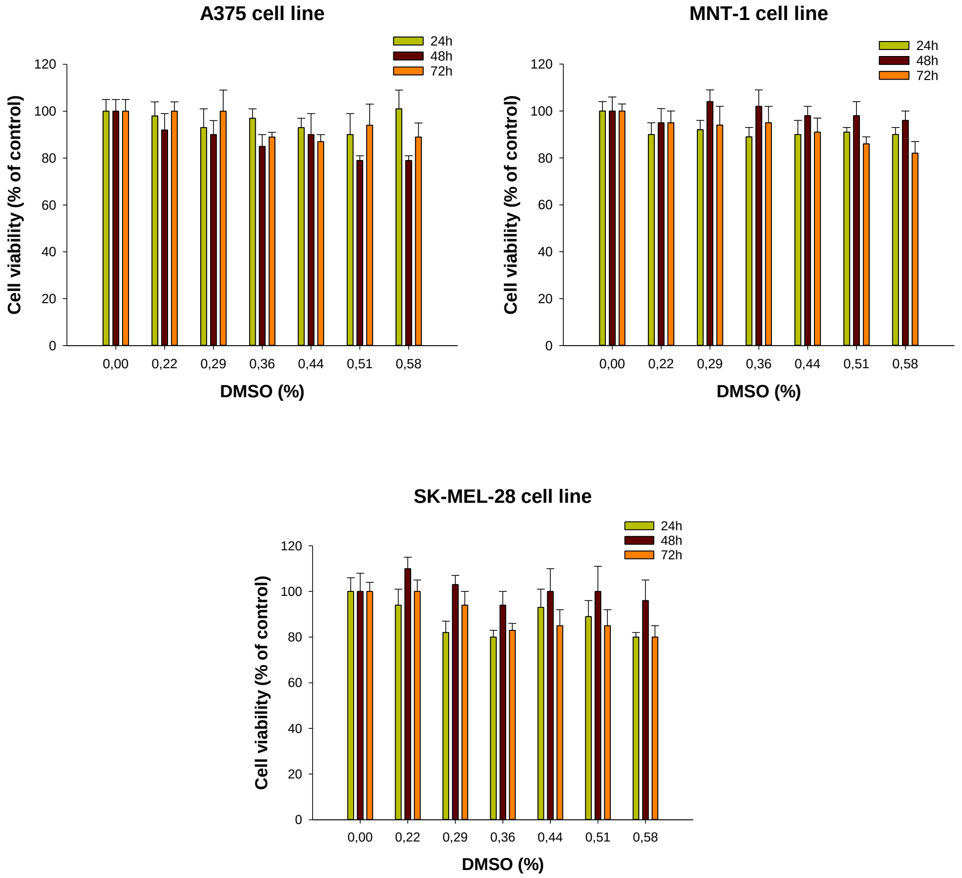

Figure S8. Effect of DMSO on cell viability of A375 cell line, MNT-1 cell line, and SK-MEL-28 cell lines. Cell viability was evaluated using MTT assay after 24, 48, and 72 h of exposure. Results are presented as mean ± standard deviation (SD).

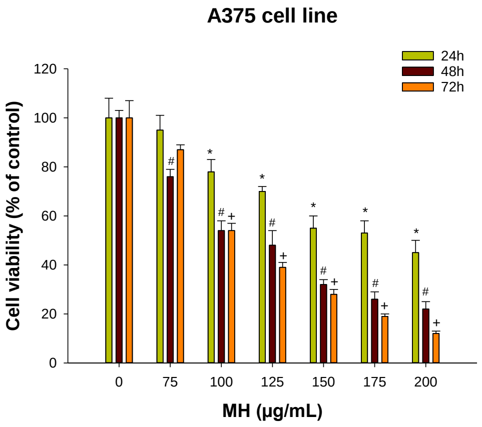

(a)

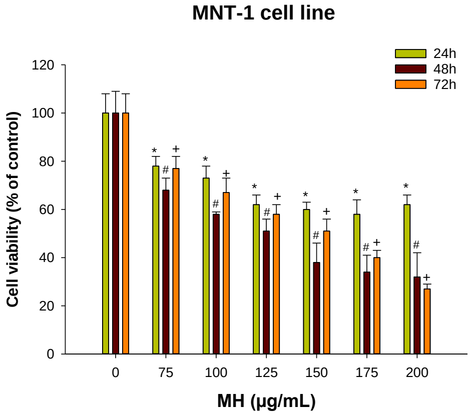

(b)

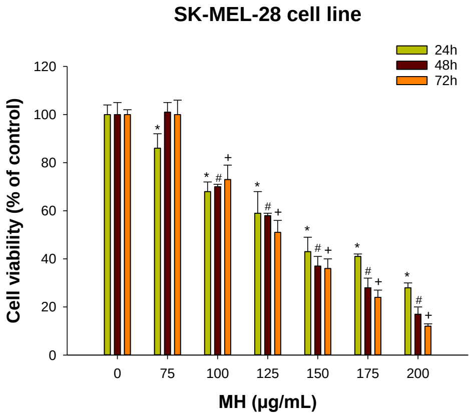

(c)

Figure S9. Effect of morin hydrate (MH) (0-200 μg/mL) on cell viability of (a) A375 cell line; (b) MNT-1 cell line; and (c) SK-MEL-28 cell line. Cell viability was evaluated using MTT assay after 24, 48, and 72 h of exposure, using the same density (20 000 cells/mL). Results are presented as mean ± standard deviation (SD). *, # and + indicate significant differences between control at $p < 0.05$ for 24 h, 48 h and 72 h, respectively.
